# Effect of combined intravenous–inhalation anesthesia on postoperative cognitive dysfunction after laparoscopic radical resection of cervical cancer: A protocol for systematic review and meta-analysis

**DOI:** 10.1097/MD.0000000000023124

**Published:** 2020-11-06

**Authors:** Ying Wang, Meihua Cao, Guofen Cao, Yujie Liu, Ying Zhang

**Affiliations:** a Department of Surgical Anaesthesia, Second Affiliated Hospital Oetianjin University of TCM, Tianjin; b Department of Nursing, Dongtai Hospital Affiliated to Nantong University, Dongtai, Jiangsu; c Department of Gynaecology Third Ward, Harbin Medical University Cancer Hospital, Harbin, Heilongjiang; d Department of Gynaecology, Second Affiliated Hospital Oetianjin University of TCM, Tianjin, China.

**Keywords:** cervical cancer, inhalation anesthesia, intravenous anesthesia, laparoscopic radical resection, meta-analysis, postoperative cognitive dysfunction, protocol

## Abstract

**Objective:**

To evaluate the effect of combined intravenous–inhalation anesthesia (CIVIA) on postoperative cognitive dysfunction (POCD) after laparoscopic radical resection of cervical cancer.

**Methods:**

By using a predefined standardized study protocol, we conducted a systematic review of randomized controlled trials (RCTs) with meta-analysis, searching the following data bases: PubMed, Embase, Web of Science, and Cochrane Library.

**Results:**

This systematic review evaluated the effect of CIVIA on POCD after laparoscopic radical resection of cervical cancer.

**Conclusion:**

This systematic review provided up-to-date evidence to evaluate the effect of CIVIA on POCD after laparoscopic radical resection of cervical cancer.

**OSF Registration number:**

DOI 10.17605/OSF.IO/82FNA.

## 1 Introduction

As a common malignant tumor in clinical gynecology, cervical cancer is one of the main malignant tumors that affect the health of women at their childbearing ages.^[1]^ Surgery is one of the most important treatments for cervical cancer, and laparoscopic minimally invasive radical resection of cervical cancer has gradually become the latest trend for the treatment of these diseases.^[2]^ As one of the most common perioperative complications, postoperative cognitive dysfunction (POCD) has serious adverse effects on the prognosis of patients.^[3]^ There is a close relationship between the depth of anesthesia and the incidence of POCD.^[4]^ Therefore, it is particularly important to choose a reasonable and effective way of anesthesia.

Intravenous anesthesia alone is induced quickly and does not stimulate the respiratory tract of the patient.^[5]^ However, this method also has some shortcomings. The metabolic rate of some drugs is slow, and after stopping the application of narcotic drugs, the recovery period of patients is longer without the use of antagonists.^[6]^ Combined intravenous–inhalation anesthesia (CIVIA) refers to a combination of intravenous anesthesia and inhalation anesthesia.^[7]^ Intravenous anesthetics take effect quickly and have no stimulation to the respiratory tract, so they are mostly applied for anesthesia induction.^[8]^ Inhaled anesthetics can effectively control the depth of anesthesia, so as to accelerate the recovery of postoperative consciousness, and is often used to maintain anesthesia.

At present, after laparoscopic radical resection of cervical cancer, the effect of intravenous inhalation combined anesthesia on cognitive function is controversial. Therefore, this study carried out a meta-analysis to provide reference evidence for clinical decision-making.

## 2 Methods

We conducted this work following preferred reporting items for systematic reviews and meta-analysis protocols (PRISMA-P) statement guidelines.^[9]^ This work was registered at OSF, and the registration number for this study is DOI 10.17605/OSF.IO/82FNA.

### 2.1 Inclusion criteria for study selection

#### 2.1.1 Types of studies

All randomized controlled trials (RCTs) of intravenous anesthesia combined with inhalation anesthesia on POCD after laparoscopic radical resection of Cervical Cancer were included without language restriction. Observational studies, conference abstracts, animal studies, data which was incomplete or contains obvious errors, and letters were all be excluded.

#### 2.1.2 Types of participants

##### 2.1.2.1 Inclusion

(1) Women, aged over 18, who underwent laparoscopic radical hysterectomy for cervical cancer.
(2) The mental state of patients is good, and the language, cognition and communication ability are normal.
(3) Patient have no history of drug allergy.
(4) Patients can tolerate surgery.

##### 2.1.2.2 Exclusion

(1) Serious diseases in blood, respiratory and circulatory system.
(2) Serious pathological changes in heart, kidney, liver, lung and other organs.
(3) Mental, cognitive and language disorders.
(4) Malignant tumors in other parts of the patient.
(5) Contraindications for laparoscopic surgery.

#### 2.1.3 Types of interventions

##### 2.1.3.1 Experimental interventions

Intravenous anesthesia combined with inhalation anesthesia

##### 2.1.3.2 Control interventions

Intravenous anesthesia

#### 2.1.4 Types of outcome measures

##### 2.1.4.1 Primary outcomes

Any score of the cognitive impairment scale includes Cognitive Failure Questionnaire (CFQ),^[10]^ Montreal Cognitive Assessment form (MoCA),^[11]^ Mini-Mental State Examination (MMSE),^[12]^ etc.

##### 2.1.4.2 Secondary outcomes

Since mood disturbance is related to POCD,^[13]^ we also assessed the mood status of patients by using Geriatric Depression Scale (GDS)^[14]^ or Zung self-rating depression scale (SDS)^[15]^ or Zung's self-rating anxiety scale (SAS),^[16]^ all of which were frequently applied as screening tools to assess mood disturbance.

### 2.2 Search methods for the identification of studies

We searched the following electronic database: PubMed, Embase, Web of Science, and Cochrane Library. We developed a search strategy that combines the following MeSH terms or keywords intravenous anesthesia, inhalation anesthesia, POCD, laparoscopic radical resection, cervical cancer, postoperative cognitive function, postoperative cognitive impairment, general anesthesia, etc. Table 1 displays the complete search strategy.

**Table 1:** Search strategy in PubMed database.

Number	Search terms
1	Anesthesia, Intravenous[MeSH]
2	Anesthesias, Intravenous[Title/Abstract]
3	Intravenous Anesthesia[Title/Abstract]
4	Intravenous Anesthesias[Title/Abstract]
5	or/1–4
6	Anesthesia, Inhalation[MeSH]
7	Insufflation Anesthesia[Title/Abstract]
8	Anesthesia, Insufflation[Title/Abstract]
9	Inhalation Anesthesia[Title/Abstract]
10	or/6–9
11	Laparoscopy[MeSH]
12	Celioscopy[Title/Abstract]
13	Laparoscopic Surgical Procedures[Title/Abstract]
14	Peritoneoscopy[Title/Abstract]
15	Surgical Procedures, Laparoscopic[Title/Abstract]
16	Laparoscopic Surgery[Title/Abstract]
17	Laparoscopic Surgical Procedure[Title/Abstract]
18	Procedure, Laparoscopic Surgical[Title/Abstract]
19	Procedures, Laparoscopic Surgical[Title/Abstract]
20	Surgery, Laparoscopic[Title/Abstract]
21	Surgical Procedure, Laparoscopic[Title/Abstract]
22	Celioscopies[Title/Abstract]
23	Laparoscopic Surgeries[Title/Abstract]
24	Laparoscopies[Title/Abstract]
25	Peritoneoscopies[Title/Abstract]
26	Surgeries, Laparoscopic[Title/Abstract]
27	or/11-26
28	Uterine Cervical Neoplasms[MeSH]
29	Cancer of Cervix[Title/Abstract]
30	Cancer of the Cervix[Title/Abstract]
31	Cancer of the Uterine Cervix[Title/Abstract]
32	Cervical Cancer[Title/Abstract]
33	Cervical Neoplasms[Title/Abstract]
34	Cervix Cancer[Title/Abstract]
35	Cervix Neoplasms[Title/Abstract]
36	Neoplasms, Cervical[Title/Abstract]
37	Neoplasms, Cervix[Title/Abstract]
38	Uterine Cervical Cancer[Title/Abstract]
39	Cancer, Cervix[Title/Abstract]
40	Cancer, Uterine Cervical[Title/Abstract]
41	Cancers, Cervix[Title/Abstract]
42	Cancers, Uterine Cervical[Title/Abstract]
43	Cervical Cancer, Uterine[Title/Abstract]
44	Cervical Cancers, Uterine[Title/Abstract]
45	Cervical Neoplasm[Title/Abstract]
46	Cervical Neoplasm, Uterine[Title/Abstract]
47	Cervical Neoplasms, Uterine[Title/Abstract]
48	Cervix Neoplasm[Title/Abstract]
49	Neoplasm, Cervical[Title/Abstract]
50	Neoplasm, Cervix[Title/Abstract]
51	Neoplasm, Uterine Cervical[Title/Abstract]
52	Neoplasms, Uterine Cervical[Title/Abstract]
53	Uterine Cervical Cancers[Title/Abstract]
54	Uterine Cervical Neoplasm[Title/Abstract]
55	or/28–54
56	Delirium, Dementia, Amnestic, Cognitive Disorders[MeSH]
57	Clerambault Syndrome[Title/Abstract]
58	Kandinsky Syndrome[Title/Abstract]
59	Mental Disorders, Organic[Title/Abstract]
60	Organic Brain Syndrome, Nonpsychotic[Title/Abstract]
61	Organic Mental Disorders[Title/Abstract]
62	Organic Mental Disorders, Psychotic[Title/Abstract]
63	Psychoses, Traumatic[Title/Abstract]
64	Nonpsychotic Organic Brain Syndrome[Title/Abstract]
65	Disorders, Organic Mental[Title/Abstract]
66	Mental Disorder, Organic[Title/Abstract]
67	Organic Mental Disorder[Title/Abstract]
68	Traumatic Psychoses[Title/Abstract]
69	Cognitive Dysfunction [Title/Abstract]
70	or/56–70
71	5 and 10 and 27 and 55 and 70

### 2.3 Data collection and analysis

#### 2.3.1 Selection of studies

**Figure 1:**
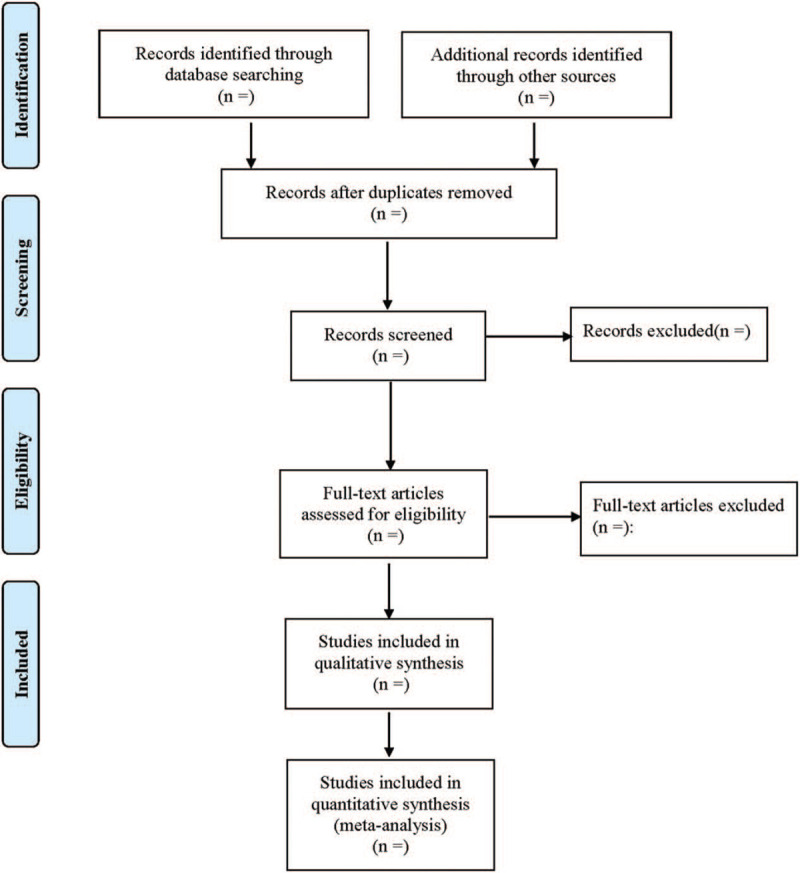
Flow diagram of study selection process.

EndNote X8 was adopted for document management. The 2 researchers independently sifted through literatures, extract data, and review their accuracy. In the aspect of literature screening, we first read the title and abstract of the research retrieved in the process of retrieval, and then read the full texts that are identified as related research. The 2 researchers must reach an agreement to decide whether to include a text, and apparently irrelevant literatures were excluded immediately. If there is any objection, a third researcher would be consulted to assist in making the judgment. Excel 2019 was used to create a data extraction table to extract relevant data. The research selection process is shown in Figure 1.

#### 2.3.2 Data extraction and management

The 2 reviewers independently extracted relevant data from qualified studies. If there was any objection, they would discuss and resolve them with the third reviewer. The data extracted from the study mainly included the first author, the year of publication, the location of the study, the baseline characteristics of participants, the total number of participants, age, detailed drug dosage, follow-up time, and cognitive function before or after operation, or the score of the scale for evaluating emotional disorders before and after surgery, etc.

### 2.4 Risk of bias assessment

The bias risk tools provided by the Cochrane library were used to assess the quality of included RCTs. We evaluated RCTs in 6 aspects, including random sequence generation, allocation hiding, blindness of participants and personnel, blindness of outcome evaluators, incomplete outcome data, selective reporting, and other sources of bias. The quality of the study was classified as “high”, “ unclear ” or “low” bias risk.

### 2.5 Quantitative data synthesis and statistical methods

#### 2.5.1 Quantitative data synthesis

RevMan 5.3 (provided by the Cochrane Collaboration) and STATA 14.0 (STATA Corporation, College Station, TX, USA) software were utilized for the statistical analysis. Standard mean difference with 95% confidence intervals will be used as continuous data.

#### 2.5.2 Assessment of heterogeneity

*Q* test was used to qualitatively determine inter-study heterogeneity. If *P* ≥ .1, it would indicate that there is no inter-study heterogeneity. However, if *P* < .1, it would reveal that there is inter-study heterogeneity present. At the same time, I^2^ statistic was used to quantitatively evaluate the inter-study heterogeneity. If *I*^2^ ≤ 50%, heterogeneity was considered to be low, and the fixed-effect model was adopted for application. Conversely, if *I*^2^ > 50%, it proved that there is significant heterogeneity, in which case the source of the heterogeneity would be explored through a subgroup or sensitivity analysis. If there was no obvious clinical or methodological heterogeneity, it would be considered as statistical heterogeneity, and the random-effects model was used for the analysis instead. Descriptive analysis was applied if there is significant clinical heterogeneity between the 2 groups and subgroup analysis was not available.

#### 2.5.3 Assessment of reporting biases

If sufficient studies were included (more than 10 studies),^[17]^ publication bias was estimated by a funnel plot analysis.

#### 2.5.4 Subgroup analysis

Subgroup analyses were carried out based on drug types, patient age, and study quality.

#### 2.5.5 Sensitivity analysis

In order to test the stability of the results, we applied 1 elimination method for sensitivity analysis.

#### 2.5.6 Ethics and dissemination

This systematic review could not require ethical approval, because there was no data used in our study and was linked to individual patients. The results were disseminated only in peer-reviewed publications.

## 3 Discussion

POCD is a postoperative complication of central nervous system in patients, and its main manifestations include anxiety, insanity, memory impairment, and even personality changes. Personality, cognitive ability, social ability and skills of the patients changed after operation and mainly degeneration.^[18]^ Mild POCD: Patients with mild memory impairment, mild cognitive impairment, response to instructions but slow response. Moderate POCD: Patients with severe memory loss and amnesia syndrome. Severe POCD: Patients with severe memory impairment, dementia, personality changes, and loss of judgment and language expression ability.^[19]^ POCD affects the quality of patients’ life. In order to ensure the prognosis of patients and make patients awake as soon as possible, doctors must do a good job in intraoperative management. Anesthesia has an impact on the central nervous system.

CIVIA combines intravenous infusion and tracheal inhalation anesthesia, and is widely applied in laparoscopic surgery.^[20,21]^ It achieved a good anesthetic effect, but the unreasonable depth of anaesthesia can affect the cognitive function of postoperative patients. We performed a meta-analysis for the first time, which may provide reliable and convincing evidence for the effect of CIVIA on cognitive function after laparoscopic radical hysterectomy for cervical cancer. The conclusions of this review may benefit patients suffering from laparoscopic radical resection of cervical cancer as well as clinicians and researchers. If this agreement needs to be amended, we will provide the date of each amendment, the statement of the amendment and the corresponding reasons.

## Author contributions

**Conceptualization:** Ying Wang and Guofen Cao

**Data collection:** Yujie Liu

**Funding acquisition:** Meihua Cao.

**Funding support:** Meihua Cao

**Investigation:** Ying Zhang.

**Literature retrieval:** Ying Zhang

**Software operating:** Yujie Liu

**Software:** Yujie Liu.

**Supervision:** Guofen Cao.

**Writing – original draft:** Guofen Cao, Ying Wang, Meihua Cao.

**Writing – review & editing:** Ying Wang and Guofen Cao
